# 3D-Printing for Transformation Optics in Electromagnetic High-Frequency Lens Applications

**DOI:** 10.3390/ma13122700

**Published:** 2020-06-13

**Authors:** Jose-Manuel Poyanco, Francisco Pizarro, Eva Rajo-Iglesias

**Affiliations:** 1 Escuela de Ingeniería Eléctrica, Pontificia Universidad Católica de Valparaíso, 2362804 Valparaíso, Chile; jose-manuel.poyanco.a@mail.pucv.cl; 2 Department of Signal Theory and Communications, University Carlos III of Madrid, 28911 Leganés, Spain; eva.rajo@uc3m.es

**Keywords:** transformation optics, 3D-printing, millimeter-wave lens

## Abstract

This article presents the design, construction and analysis of a 3D-printed transformed hyperbolic flat lens working on the 30 GHz band. The transformed lens was printed using only one ABS dielectric filament of relative permittivity of 12, varying the infill percentage of each transformed lens section in order to achieve the permittivity values obtained with the transformation optics. The 3D-printed hyperbolic transformed lens exhibits good radiation performance compared to the original canonical lens.

## 1. Introduction

In the last years, transformation optics has been an important subject of study for the scientific community. This tool, initially proposed in [1,2] is a method that allows the definition of a modification of the dielectric constant ε and the permeability μ into another coordinate system by keeping the same electromagnetic properties. This tool allows to transform any electromagnetic device into a different equivalent, having the same electromagnetic characteristics but with a different morphology. This has been the kickoff of many interesting applications [3,4,5], specially in electromagnetic high-frequency topologies [6,7]. One of the main applications of this tool is oriented to improve lens designs, such as simplifying their geometry and reducing their overall size [8,9,10]. Nevertheless, the main drawback of this tool is the high cost or difficulties involved on the construction and implementation of the designed devices, mainly due to the shapes and dielectric characteristics obtained for the transformed structure.

One of the main difficulties to obtain the different dielectric constants to achieve the respective refraction indexes is the availability of dielectric constants itself. The fabrication of parts that are needed to have a specific dielectric constant value, can be a difficult task for any dielectric or ceramic manufacturer. The impossibility of achieving a desired permittivity value, either due to manufacturing or cost reasons, can force to introduce changes into the original designs to adapt them to the available permittivity values [11]. Therefore, 3D-printing is a candidate to implement this technology in order to obtain permittivity values closer to the ones estimated in the original designs. New materials available for printing, such as low-loss ABS filaments [12] and ceramics [13] make possible the easy fabrication of dielectrics with different relative permittivities and allows any shape for the manufactured structures. In addition, the reduction of the cost of the 3D-printers and their improvement in terms of precision and resolution allows the construction of models that were either too expensive or difficult to manufacture [14,15]. This combination of high precision, low-cost and new materials available has led to the implementation of several high frequency topologies [16,17,18,19,20,21,22], including 3D-printed lenses [23,24,25].

Recently, several studies have been done in relation to the use of different lenses oriented to the new generation of communications, named 5G [26,27,28,29]. This technology will use a higher part of the electromagnetic spectrum in order to achieve larger transmission bandwidths [30,31,32]. The main problem of using these frequency bands is the increase on the propagation losses, that can be compensated by the use of antenna arrays and lenses. Therefore, it can be important for the development of this technology the use of low-cost and rapid prototyping of possible transformed optic topologies for its application. This article presents the design, construction and assessment of a 3D-printed hyperbolic flat lens designed with transformation optics to work in the 30 GHz band. The transformation optic design is based on the work presented in [11], with the difference that this design and its transformation optic will be fully 3D-printed using PREMIX ABS dielectric filaments [12] using a low-cost 3D-printer, and operating in a higher frequency band.

This article is divided as follows: Section 2 presents the hyperbolic canonical lens, its transformed flat version and the assessment and comparison of their radiating performances. Section 3 contains the 3D-printing design and procedure of the transformed lens and finally, in Section 4 the assessment of the 3D-printed transformed hyperbolic flat lens is presented and compared with the ideal designed transformed lens.

## 2. Transformation Optics and Lens Design

**Figure 1:**
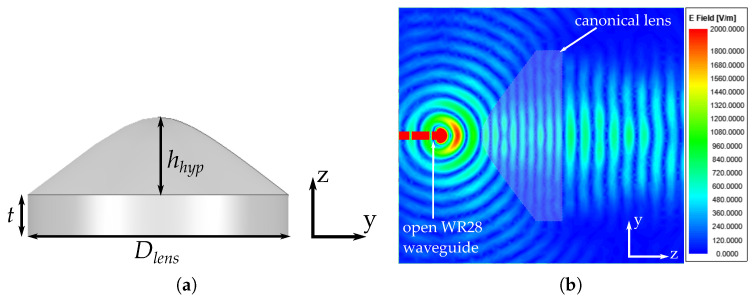
Canonical hyperbolic lens. (**a**) Dimensions. (**b**) Resulting electric field at 30 GHz.

To apply the transformation optics, a hyperbolic canonical lens was chosen. The design of this kind of lenses is well known and applied in many situations for high frequency devices [33,34]. The designed lens will work on the 30 GHz band, due to its potential applications in the next generation of communication standards [31,32]. The geometry of a canonical hyperbolic lens can be described by the Equation (1) (in spherical coordinates):

(1) $$\rho \left(\phi \right)=\frac{(n-1)f}{n\cos\phi -1}$$

where *n* is the refractive index, *f* the focal distance and ϕ the angle between the focal axis and a point in the curved shape. The design of this canonical lens follows the guidelines exposed in [33]. First, we start by defining the material and therefore the permittivity to be used for the calculation of the refractive index, as $n=\sqrt{\varepsilon_{r}\mu_{r}}$, where $\varepsilon_{r}$ is the relative permitivitty of the chosen material and $\mu_{r}$ is the relative permeability of the material (which in our case, $\mu_{r}=1$). For this lens, we have chosen a dielectric material with relative permittivity $\varepsilon_{r}=$ 2.53. The other important parameter to define is the focal distance, that was set at 19 mm. The diameter of the lens was set at 7.6 $\lambda_{0}$ at 30 GHz. The resulting designed canonical hyperbolic lens and the generated electric field are shown in Figure 1. The lens is divided in two sections, one cylindrical with height t= 12 mm and one hyperbolic section with $h_{hyp}=$ 24.3 mm, while the resulting lens diameter is $D_{lens}=$ 76 mm. Regarding the generated electric field, the lens is fed with an open WR-28 standard waveguide at a focal distance of 19 mm with a frequency $f_{0}=$ 30 GHz and simulated with the full-wave simulation software ANSYS HFSS [35]. From the electric field result exposed in Figure 1b we can see that the incident field on the lens is transformed into a plane wave when coming from the lens, as expected from this canonical lens. In addition we can confirm that the lens is well designed due to the distribution of the field inside the lens in terms of low internal reflections and low backwards reflections.

**Figure 2:**
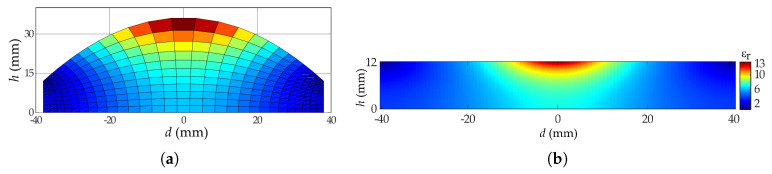
Transformation optics for the canonical hyperbolic lens. (**a**) Grid of the hyperbolic lens (colors indicate the surface difference between grids). (**b**) Transformed flat lens with relative permittivity ($\varepsilon_{r}$) distribution.

One of the main problems of constructing hyperbolic lenses is related to its manufacture difficulties with good accuracy. Any deformation on the lens will affect on its overall efficiency and for this reason, the flattening of this kind of lenses can solve that manufacturing problem. For that, we will apply a quasi-conformal transformation optic to this lens based on the work presented in [11]. Applying this methodology, we obtain a planar lens with a variable permittivity. We perform a mesh into the original canonical lens, and then mapped it into a planar structure. Each point of the planar lens has a specific permitivitty, related with the area of each unit cell of the hyperbolic lens. The grid of the canonical hyperbolic lens and its respective transformed version are shown in Figure 2. We can see that in order to transform the canonical hyperbolic lens into a flat lens working on the same frequency band, using the selected grid structure, we need to implement several sections with different shapes and permittivity values. This particular lens has permittivity values that can go up to $\varepsilon_{r}=$ 12.6.

**Figure 3:**
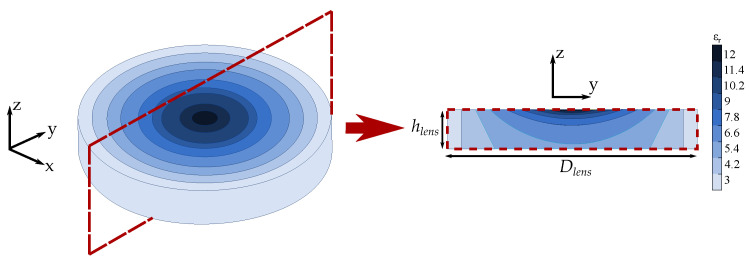
Transformed hyperbolic lens with cut view and section permittivity values.

To implement this lens using 3D-printing, we have to take into account the available materials that can reach the permittivity values given by the transformation optic. For that, we will use ABS filaments from PREMIX, which have several available permittivity values, and are characterized in high frequency, with low losses [12]. When this work was done, the maximum permittivity value available for these ABS filaments was $\varepsilon_{r}=$ 12. Therefore, and in order to implement the transformed lens, we have to truncate the maximum permittivity value of the lens from $\varepsilon_{r}=$ 12.6 to $\varepsilon_{r}=$ 12. The lens consists of nine different permittivity sections, with permittivity values that go from $\varepsilon_{r}=$ 3 to $\varepsilon_{r}=$ 12, being possible to be printed with the available ABS commercial filaments. In Figure 3 the transformed hyperbolic flat lens with the corresponding section shapes and permittivity values is shown. The dimensions of the lens are $D_{lens}=76$ mm and $h_{lens}=12$ mm, which are the same dimensions of the rear-planar part of the reference canonical hyperbolic lens.

**Figure 4:**
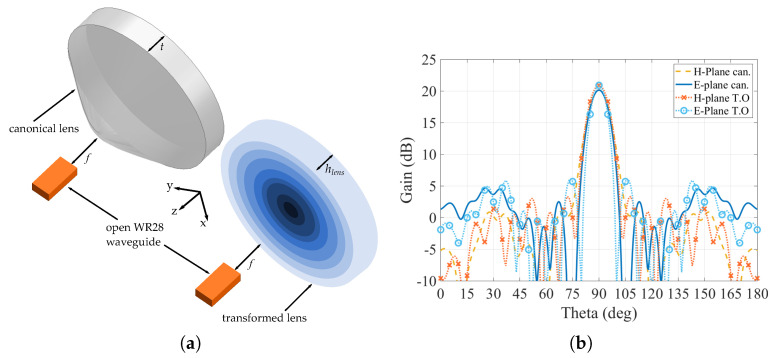
Hyperbolic lenses under test. (**a**) Canonical and transformed lenses with their feeding open waveguide. (**b**) Gain radiation pattern (E-Plane and H-Plane) of the canonical hyperbolic lens (can.) and the hyperbolic transformed flat lens (T.O).

As the transformed lens was modified in order to fit the available filaments, it is necessary to assess if this modification has an important effect on the radiating parameters of the lens. For this evaluation, we will compare the simulated gain radiation patterns generated by the canonical hyperbolic lens at 30 GHz with the gain radiation pattern generated by the modified transformed flat lens at the same frequency. Both lenses are fed with an open WR-28 standard waveguide placed at a focal distance f= 19 mm for the canonical lens and f= 43.3 mm for the flat-transformed one, which corresponds to the initial focal distance, plus the flattened part of the canonical lens $h_{hyp}$ in order to make the equivalent to the feeding point. The simulations are done using the full-wave simulation software ANSYS HFSS. The lenses under test and the resulting radiation patterns are shown in Figure 4. From the simulated results of the gain radiation pattern in both H-plane and E-plane of the structure we can see that there are no significant variations on the overall performance of the transformed lens in comparison with the canonical, having both a maximum gain value of around 21 dB. The only noticeable difference is just a slight increase on the sidelobe levels. Therefore, the simulated results confirm that the transformed lens works perfectly at the selected frequency, without significant differences with respect to the canonical one.

## 3. 3D-Printing of Transformed Lens

**Figure 5:**
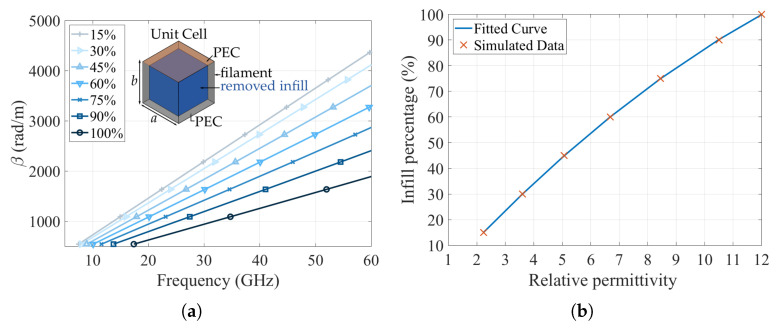
Unit cell analysis for different infill percentage values. (**a**) Dispersion diagram as a function of the infill percentage for ABS1200 filament. (**b**) Corresponding permittivity value as a function of the infill percentage for the ABS1200 filament.

One of the reasons to implement transformation optics using 3D-printing is the reduction of the overall cost of the prototypes and their fabrication times. If we analyze the resulting transformed lens in this example, we have several permittivity values that are not easy to find in commercial dielectrics available on the market for high frequency applications. In a further analysis, there are not even ABS filaments characterized in high frequency with the needed values for our transformed lens to be printed directly. However, there are manufacturing techniques that make possible the fabrication using 3D-printing of non-available permittivity values. One of these techniques is to vary the infill percentage of a section in order to achieve lower permittivity values [24,36]. There are many techniques to asses a permittivity value in simulation created with an infill percentage of a 3D-printed structure. One method is to create a substrate that represents the infill percentage and estimate the effective permittivity with the resonance frequency of resonant structure [22,37], or the Nicolson-Ross-Weir method, by placing a substrate with the required infill inside a waveguide section, and retrieving the permittivity value with the scattering parameters [38,39]. The problem of these two methods is that we need to generate the sample under test with the approximate infill as close as the printer will do, and proceed with a high-consuming computational resources simulation. One technique that demonstrates to be useful and low-consuming in terms of computational resources is to create sub-lambda periodic unit cell to obtain the dispersion diagram (propagation constant β as function of the frequency) generated by the periodic structure. The unit cell itself consists of two parallel plates made of perfect conductor material (PEC) and in between, the material with the permittivity to be assessed is inserted. Then, a percentage of the material is retired from the structure, simulating a lower infill and obtaining a new dispersion diagram [36]. This sub-lambda periodic structure represents the grid generated by the 3D-printing in a section for different infill percentages. Figure 5a shows the dispersion diagram with the unit cell for different infills. In the calculation of these dispersion diagrams, we only consider the first propagation mode (a TEM in the parallel plate structure) and lossless materials. As a consequence, the solver gives directly the phase constant. The unit cell size is a=b=1.66 mm, which corresponds to $\lambda_{0}/6$ at 30 GHz and the filament chosen for this analysis was the ABS1200 of PREMIX with $\varepsilon_{r}=$ 12.

Once we have the propagation constant values for each infill percentage of the structure, and knowing that the material is within a parallel plate structure, the relative permittivity values can be easily calculated by the relation $\beta =\omega \sqrt{\mu \varepsilon }$ [40]. The relative permittivity results obtained from the different infills of the ABS1200 filament are shown in Figure 5b. Using the obtained data, we can obtain a fitted curve that allows us to estimate the permittivity as a function of the infill percentage. The fitted curve for the relative permittivity $\varepsilon_{r}$, where the variable *x* is the infill percentage, is shown in Equation (2):

(2) $$ABS1200\left(\varepsilon_{r}\right)=-0.002684x^{2}+0.1251x-0.1159$$

It is possible to repeat this process for all the commercial available filaments. However, and with the perspective of an easy manufacturing, we will use only one filament to print the whole transformed lens. This strategy will avoid the need to pause the printing process and change filaments, which can induce a printing error, as this process has to be repeated for every section that will need a new filament. Under this perspective, the only filament that can be used to fulfill the transformed lens permittivities is the $\varepsilon_{r}=$ 12 filament. In Table 1 the relative permittivity values of each section of the transformed lens with its corresponding infill percentage using one filament of $\varepsilon_{r}=$12 are shown.

With the material and fabrication method selected, it is important to know the fabrication limits in terms of resolution and temperatures that the 3D printer can achieve. The printer that is used to construct the transformed lens is a custom 3D-printer made by Ocular3D [41]. The most relevant printer characteristics are listed on Table 2. To notice that the printer parameters are suitable for printing the selected material [12].

**Figure 6:**
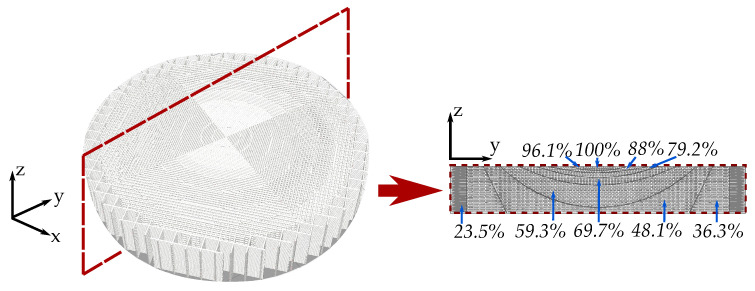
Transformed hyperbolic lens printed with one filament of $\varepsilon_{r}=$ 12 and the corresponding infill percentages for each permittivity value.

For the lens fabrication, first we export our design from the full-wave simulation software to a conventional 3D-printing software, such as Ultimaker CURA [43]. Once the printer and material profile are loaded, we select each section of different permittivity and assign the infill percentage that corresponds to the desired relative permittivity. Figure 6 shows the CURA generated transformed hyperbolic lens printed with one filament of $\varepsilon_{r}=$ 12 and the cross-section with the corresponding infill percentages for each permittivity value of the transformation optic.

**Figure 7:**
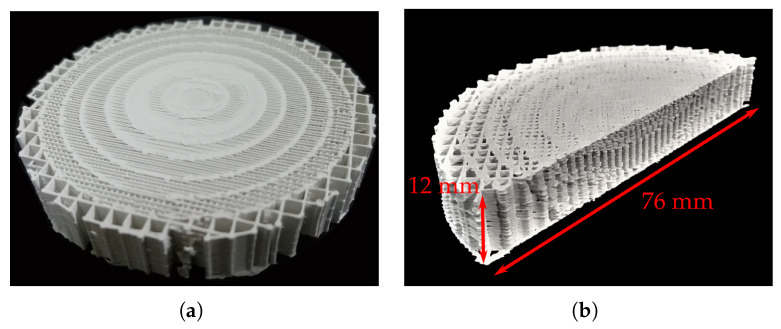
3D-printed transformed hyperbolic flat lens. (**a**) 3D-printed prototype. (**b**) Cut-view over the 3D-printed transformation optic.

Once the CURA design is ready, the ABS1200 filament is printed using the printing parameters shown in Table 3. Figure 7 shows the 3D-printed transformed lens and a cut view of the printed transformation optic. We can see that the transformation optic is printed correctly, having the different sections with the corresponding infills.

## 4. 3D-Printed Transformed Lens Results

**Figure 8:**
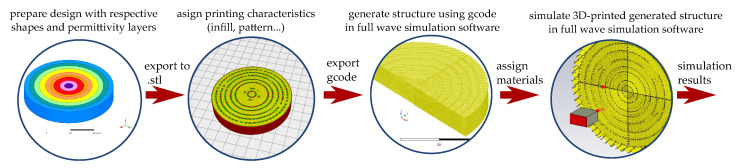
Flow diagram for full-wave simulation of 3D-printed generated structures.

Now that we have the possibility to construct the transformed lens using conventional 3D-printing, we need to assess the behavior of the 3D transformed structure. One procedure to evaluate the 3D-printed transformation optic we use is shown in Figure 8. First, and after the design of the transformed structure, we prepare the 3D design using any 3D CAD program. In this step we divide the sections of different permittivities, i.e., we do the discretization of the structure. Once the design is complete, we export into a format, for example .stl, that a commercial 3D-printing software (e.g., Ultimaker CURA) can read. Once the design is imported, using the software we assign the printing pattern and infill percentage for each section of the transformed structure, and the type of filament and its characteristics. When all the infill percentages are defined, we export the design into a gcode file, that can be used for printing the transformed lens as-is. The gcode file contains the actual instructions for the printer, in terms of filament dimensions, positions of each deposition, and direction of the extrusion process. As the gcode has the information of position of each filament deposition, as well as its dimensions and shape (or track), it can be easily translated into another 3D-CAD software, or directly into a full-wave simulation software such as ANSYS HFSS or CST Microwave Studio [44], assuring to obtain a structure made of the filament as the printer will construct it during the printing process. Therefore, this new generated structure has the actual filament distribution on the volume of the transformed structure. In other terms, the actual printed model is available to be analyzed. The final step is to assign the material characteristics to the structure. In this case, as the transformed lens is constructed by one material, the electrical characteristics of the PREPERM ABS1200 are assigned to the whole structure ($\varepsilon_{r}=$ 12, tanδ= 0.004 [12]). Finally the structure is simulated and analyzed with the full-wave simulation software.

**Figure 9:**
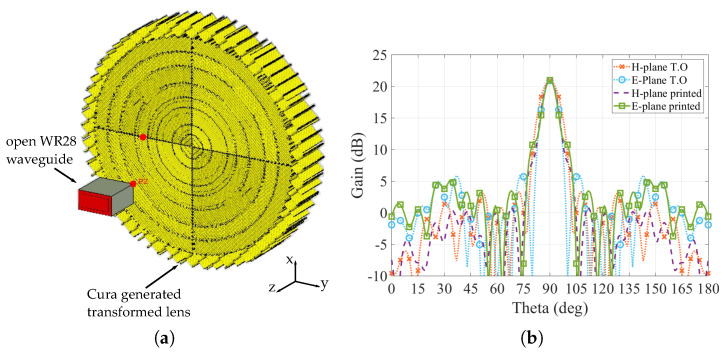
Cura generated 3D-printed transformed hyperbolic lens. (**a**) Simulation setup. (**b**) Gain radiation pattern of 3D-printed lens compared to the solid transformed lens.

For the assessment of the printed transformed lens, the as-is generated structure from the 3D-printer gcode is imported into CST Microwave Studio using a previous translation of the gcode with MatLab. The parameters to be evaluated are the gain radiation pattern and the electric field distribution of the lens, and compare them with the obtained simulation results of the solid transformed lens (Figure 3 and Figure 4b). The printed lens is fed with an open WR-28 waveguide, using a frequency $f_{0}=$ 30 GHz, and placed at the same focal distance that was used in the results shown in Figure 4b. The simulated printed transformed lens and the comparison of the gain radiation patterns of the 3D-printed lens and the solid transformed lens are shown in Figure 9.

From the results, we can see that there is a good match between the solid previous simulated structure and the 3D-printed simulated structure. To notice that the solid structure is the transformed structure made by solid sections, with each permittivity value of the sections assigned on the software (i.e., 9 sections with 9 different materials), while the 3D-printed simulated lens is composed by just one material (ABS1200), obtaining the nine different permittivity values by using the estimated infill percentage in the construction of the lens and with the corresponding discretization. This good agreement between the gain radiation patterns confirms that with the calculated infill percentages, and using them into a standard 3D-printing software, the resulting structure will have the expected behavior in terms of resulting permittivity values.

**Figure 10:**
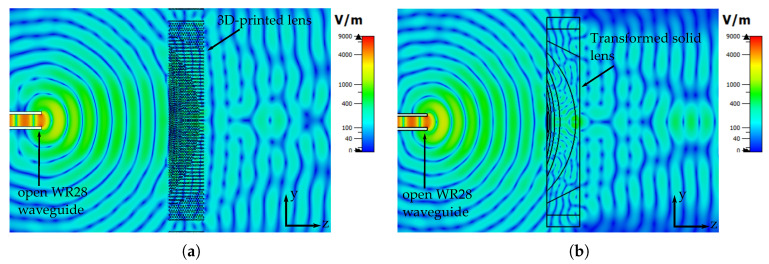
Simulated electric field at 30 GHz (**a**) Transformed 3D-printed lens. (**b**) Transformed solid lens.

One final assessment that is done is the comparison of the generated electric fields of the lenses. In Figure 10 a representation of the simulated electric fields at 30 GHz of the solid transformed lens and the 3D-printed transformed lens is shown. We can observe that both lenses can successfully transform the generated wave from the waveguide into a plane wave. In addition there are no notorious internal reflections inside the lens or important backscatters that can occurs if the lens is not designed correctly.

## 5. Conclusions

This article presents a fully 3D-printed version of a transformed hyperbolic flat lens designed for the 30 GHz band. The lens was successfully printed using one material, varying the infill percentages to achieve the different permittivity values required by the transformation optics results. The printed lens as-is was compared to an ideal version, showing a good agreement between the radiating characteristics. This study confirms the possibility of manufacturing complex structures obtained from transformation optics, in terms of shapes and permittivity values. To notice that by constructing this structure using an additive manufacturing low cost process, in addition to the simple use of just one dielectric filament, also confirms that this kind of complex structures can be done using a low-cost manufacturing process.

Future work will include the design and measurement of other transformed lenses, in order to assess the fabrication limits of conventional low-cost 3D-printers, as well as the use of combined materials in order to assess other parameters, such as mechanical resistance or exposure to application conditions.

## Figures and Tables

**Table 1:** Relative permittivity values and corresponding infill percentage of each section of the transformed lens, using one filament of $\varepsilon_{r}=$ 12.

Parameter	Value								
lens section ($\varepsilon_{r}$)	12	11.4	10.2	9.0	7.8	6.6	5.4	4.2	3.0
infill percentage (%)	100	96.1	88.0	79.2	69.7	59.3	48.1	36.3	23.5

**Table 2:** Ocular3D EIE-custom 3D printer technical specifications.

Printer Parameter	Value
Maximum printing volume	190 × 190 × 190 mm${}^{3}$
Axis resolution	100 μm in all axis (*xyz*)
Hot-end model	Two E3DV6 [42]
Hot-end T${}^{\circ }$ range	120 to 280 °C
Platform maximum T°	100 °C

**Table 3:** 3D-printer settings for ABS1200 printing.

Printing Setting	Value
Layer height	0.3 mm
Wall Thickness	no wall
Infill pattern	automatic
Printing temperature	260 °C
Build plate temperature	80 °C
Build plate adhesion type	raft
Printing speed	15 mm/s
Travel speed	120 mm/s
Initial layer speed	12.5 mm/s
Flow	105%

## References

[1] Leonhardt U. (2006). Optical Conformal Mapping. Science.

[2] Pendry J.B., Schurig D., Smith D.R. (2006). Controlling Electromagnetic Fields. Science.

[3] Smolyaninova V.N., Smolyaninov I.I., Kildishev A.V., Shalaev V.M. (2010). Broadband Transformation Optics Devices. Materials.

[4] Smolyaninova V., Lahneman D., Adams T., Gresock T., Zander K., Jensen C., Smolyaninov I. (2015). Experimental Demonstration of Luneburg Waveguides. Photonics.

[5] Hong J.S., Cheng W.M., Yang M.C., Shiu R.C., Lan Y.C., Chen K.R. (2018). Enhancing Efficiency of Electromagnetic Simulation in Time Domain with Transformation Optics. Appl. Sci..

[6] Kwon D.H., Werner D.H. (2008). Polarization splitter and polarization rotator designs based on transformation optics. Opt. Express.

[7] Pendry J.B., Aubry A., Smith D.R., Maier S.A. (2012). Transformation Optics and Subwavelength Control of Light. Science.

[8] Demetriadou A., Hao Y. (2011). Slim Luneburg lens for antenna applications. Opt. Express.

[9] Kwon D.H., Werner D.H. (2009). Flat focusing lens designs having minimized reflection based on coordinate transformation techniques. Opt. Express.

[10] Quevedo-Teruel O., Tang W., Hao Y. (2012). Isotropic and nondispersive planar fed Luneburg lens from Hamiltonian transformation optics. Opt. Lett..

[11] Quevedo-Teruel O., Tang W., Mitchell-Thoma R., Dyke A., Dyke H., Zhang L., Haq S., Hao Y. (2013). Transformation optics for antennas: Why limit the bandwidth with metamaterials?. Sci. Rep..

[12] Premix Preperm Website. https://www.preperm.com.

[13] 3DCERAM Website. https://3dceram.com/.

[14] Kim C., Espalin D., Liang M., Xin H., Cuaron A., Varela I., Macdonald E., Wicker R.B. (2017). 3D Printed Electronics with High Performance, Multi-Layered Electrical Interconnect. IEEE Access.

[15] Willis S. (2018). The Maker Revolution. Computer.

[16] Massaccesi A., Dassano G., Pirinoli P. (2019). Beam Scanning Capabilities of a 3D-Printed Perforated Dielectric Transmitarray. Electronics.

[17] Zhang B., Guo Y.X., Sun H., Wu Y. (2017). Metallic, 3D-Printed, K-Band-Stepped, Double-Ridged Square Horn Antennas. Appl. Sci..

[18] Lee S., Yang Y., Lee K.Y., Jung K.Y., Hwang K. (2018). Robust Design of 3D-Printed 6–18 GHz Double-Ridged TEM Horn Antenna. Appl. Sci..

[19] Chen Q., Chen X., Xu K. (2017). 3-D Printed Fabry–Pérot Resonator Antenna with Paraboloid-Shape Superstrate for Wide Gain Bandwidth. Appl. Sci..

[20] Zhong Z.P., Liang J.J., Huang G.L., Yuan T. (2018). A 3D-Printed Hybrid Water Antenna with Tunable Frequency and Beamwidth. Electronics.

[21] So K., Luk K., Chan C., Chan K. (2018). 3D Printed High Gain Complementary Dipole/Slot Antenna Array. Appl. Sci..

[22] Pizarro F., Salazar R., Rajo-Iglesias E., Rodríguez M., Fingerhuth S., Hermosilla G. (2019). Parametric Study of 3D Additive Printing Parameters Using Conductive Filaments on Microwave Topologies. IEEE Access.

[23] Pourahmadazar J., Sahebghalam S., Abazari Aghdam S., Nouri M. A Millimeter-Wave Fresnel Zone Plate Lens Design Using Perforated 3D Printing Material. Proceedings of the 2018 IEEE MTT-S International Microwave Workshop Series on Advanced Materials and Processes for RF and THz Applications (IMWS-AMP).

[24] Zhang S., Arya R.K., Pandey S., Vardaxoglou Y., Whittow W., Mittra R. (2016). 3D-printed planar graded index lenses. IET Microwaves Antennas Propag..

[25] Friel R.J., Gerling-Gerdin M., Nilsson E., Andreasson B.P. (2019). 3D Printed Radar Lenses with Anti-Reflective Structures. Designs.

[26] Quevedo-Teruel O., Miao J., Mattsson M., Algaba-Brazalez A., Johansson M., Manholm L. (2018). Glide-Symmetric Fully Metallic Luneburg Lens for 5G Communications at Ka-Band. IEEE Antennas Wirel. Propag. Lett..

[27] Kim E., Ko S., Lee Y.J., Oh J. (2018). Millimeter-Wave Tiny Lens Antenna Employing U-Shaped Filter Arrays for 5G. IEEE Antennas Wirel. Propag. Lett..

[28] Imbert M., Romeu J., Baquero-Escudero M., Martinez-Ingles M., Molina-Garcia-Pardo J., Jofre L. (2017). Assessment of LTCC-Based Dielectric Flat Lens Antennas and Switched-Beam Arrays for Future 5G Millimeter-Wave Communication Systems. IEEE Trans. Antennas Propag..

[29] Dehghani Kodnoeih M.R., Letestu Y., Sauleau R., Motta Cruz E., Doll A. (2018). Compact Folded Fresnel Zone Plate Lens Antenna for mm-Wave Communications. IEEE Antennas Wirel. Propag. Lett..

[30] Rappaport T.S., Sun S., Mayzus R., Zhao H., Azar Y., Wang K., Wong G.N., Schulz J.K., Samimi M., Gutierrez F. (2013). Millimeter Wave Mobile Communications for 5G Cellular: It Will Work!. IEEE Access.

[31] 3GPP Release Description; Release 15. Technical Specification (ts), 3rd Generation Partnership Project (3GPP), 2019. TR 21.915. https://www.etsi.org/committee/1418-3gpp.

[32] Al-Dulaimi A., Wang X., Chih-Lin I. (2018). Standardization: The Road to 5G. 5G Networks: Fundamental Requirements, Enabling Technologies, and Operations Management.

[33] Milligan T.A. (2005). Modern Antenna Design.

[34] Piksa P., Zvanovec S., Cerny P. (2011). Elliptic and Hyperbolic Dielectric Lens Antennas in mm-Waves. Radioengineering.

[35] ANSYS Website. https://www.ansys.com.

[36] Bjorkqvist O., Zetterstrom O., Quevedo-Teruel O. (2019). Additive Manufactured Dielectric Gutman Lens. Electron. Lett..

[37] Moolat R., Mani M., Abdulrahiman S.V., Pradeep A., Kesavath V., Pezholil M. (2020). Liquid Permittivity Sensing Using Planar Open Stub Resonator. J. Electron. Mater..

[38] Nicolson A.M., Ross G.F. (1970). Measurement of the Intrinsic Properties of Materials by Time-Domain Techniques. IEEE Trans. Instrum. Meas..

[39] Weir W.B. (1974). Automatic measurement of complex dielectric constant and permeability at microwave frequencies. Proc. IEEE.

[40] Pozar D. (2011). Microwave Engineering.

[41] Ocular 3D Website. http://ocular3d.cl/.

[42] E3D Website. https://e3d-online.com.

[43] Ultimaker Cura Website. https://ultimaker.com/.

[44] 3DS Website, CST. https://www.3ds.com/.

